# The emerging role of Piezo1 in the musculoskeletal system and disease

**DOI:** 10.7150/thno.96959

**Published:** 2024-06-24

**Authors:** Lei Lei, Zhenkang Wen, Mingde Cao, Haozhi Zhang, Samuel Ka-Kin Ling, Bruma Sai-Chuen Fu, Ling Qin, Jiankun Xu, Patrick Shu-Hang Yung

**Affiliations:** 1 Musculoskeletal Research Laboratory and Centre of Musculoskeletal Aging and Regeneration, Department of Orthopaedics and Traumatology, Faculty of Medicine, The Chinese University of Hong Kong, Hong Kong SAR, China.; 2 The Sir Yue-Kong Pao Cancer Centre, Prince of Wales Hospital, The Chinese University of Hong Kong, Hong Kong SAR, China.; 3 Joint Laboratory of Chinese Academic of Science and Hong Kong for Biomaterials, The Chinese University of Hong Kong, Hong Kong SAR, China.

**Keywords:** Piezo1, bone, muscle, cartilage, intervertebral disc

## Abstract

Piezo1, a mechanosensitive ion channel, has emerged as a key player in translating mechanical stimuli into biological signaling. Its involvement extends beyond physiological and pathological processes such as lymphatic vessel development, axon growth, vascular development, immunoregulation, and blood pressure regulation. The musculoskeletal system, responsible for structural support, movement, and homeostasis, has recently attracted attention regarding the significance of Piezo1. This review aims to provide a comprehensive summary of the current research on Piezo1 in the musculoskeletal system, highlighting its impact on bone formation, myogenesis, chondrogenesis, intervertebral disc homeostasis, tendon matrix cross-linking, and physical activity. Additionally, we explore the potential of targeting Piezo1 as a therapeutic approach for musculoskeletal disorders, including osteoporosis, muscle atrophy, intervertebral disc degeneration, and osteoarthritis.

## Introduction

Mechanotransduction enables the cells to translate mechanical forces into biochemical signals 1, thus triggering a series of biological responses. Mechanically activated ion channels, as force-sensing integral membrane proteins, can couple their structural dynamics and membrane proteins to adapt mechanical stimuli to cell plasma membrane 2. These channels are involved in various physiological processes, such as touch, hearing, proprioception, and osmoregulation 3-5. In 2010, Coste *et al.* discovered the Piezo family of mechanosensitive ion channels encoded by the *Piezo1/FAM38A* and* Piezo2/FAM38B* genes 6. Piezo1 is predominantly expressed in non-sensory tissues and responds to mechanical loading, whereas Piezo2 is located in sensory tissues and senses touch 7.

Cell membrane deformations caused by mechanical forces can activate the Piezo1 channel 8. This channel can directly convert mechanical forces, such as shear stress and osmotic pressure, into physiological changes through depolarization of excited cells or inducing cationic influx in non-excitable cells 9. Piezo1 exhibits permeability to both monovalent and divalent ions, such as Ca^2+^, Na^+^, K^+^, and Mg^2+^, with a preference of Ca^2+^. A wealth of circumstantial evidence suggests that Piezo1 expressed in most mammals is engaged in various physiological processes, such as lymphatic vessel development 7, axon growth 10, vascular development 11, immunoregulation 12, and blood pressure regulation 13, indicating the universal significance of the Piezo1 channel.

Piezo1 channel plays a crucial role in sensing mechanical stimulation and regulating cell behaviors, such as proliferation, migration, and apoptosis 14-16. For instance, acoustic radiation force can promote osteoblasts migration and proliferation by upregulating Piezo1 expression 17. It is worth noting that Piezo1 also participates in stem cell fate determination. Piezo1 is robustly expressed in stem cells and its modulation can impact differentiation outcomes 18. Piezo1 knockdown promotes astrogenesis and suppresses neurogenesis in human neural stem cells 19. In addition, Piezo1 enhances the osteogenic differentiation of mesenchymal stem cells (MSCs) via increasing BMP2 expression 20. Notably, Piezo1 is expressed stably in cells resident in bone 21, cartilage 22, skeletal muscle 23, tendon 24, intervertebral disc 25, and even their surrounding connective tissues 26. Piezo1 promotes angiogenesis to accelerate bone fracture healing 27. Besides, Piezo1 induces myotube formation by controlling Ca^2+^ influx 28, as well as enhances tendon stiffness through modifying collagen cross-linking 24. Of note, Piezo1 activation however promotes the progression of osteoarthritis (OA) 29.

**Figure 1:**
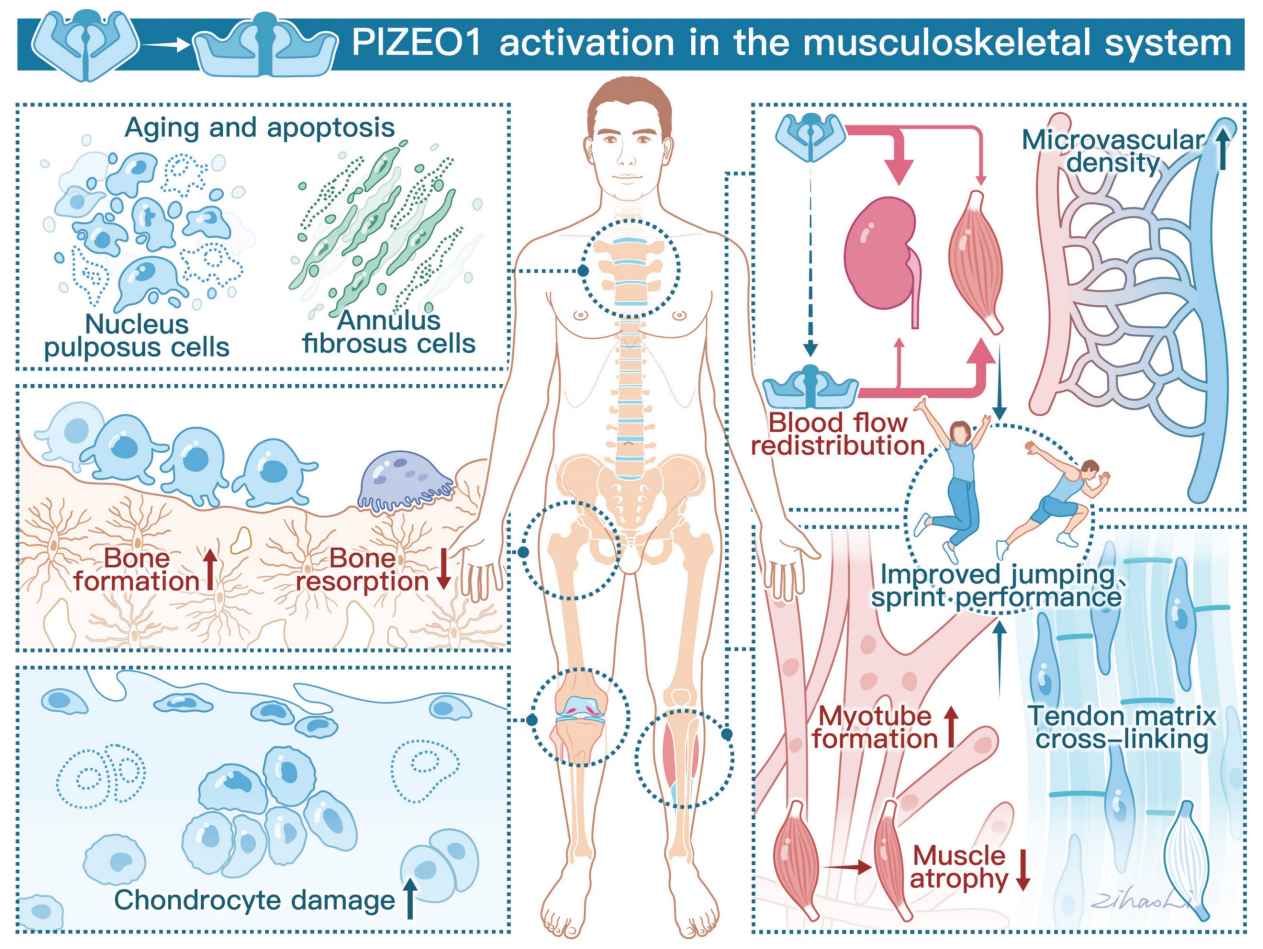
**Schematic diagram showing the crucial roles of Piezo1 in the musculoskeletal system.** The left panel demonstrates that Piezo1 activation enhances bone formation and reduces bone resorption. However, Piezo1 activation accelerates chondrocyte damage and promotes nucleus pulposus (NP) and annulus fibrosus (AF) cell senescence and apoptosis. The right panel highlights the positive effects of Piezo1 activation on physical performance. Specifically, Piezo1 activation leads to increased blood flow redistribution and microvascular density in muscles. Furthermore, Piezo1 activation promotes myotube formation and increases tendon stiffness.

Recently, two reviews 30, 31 have summarized the role of Piezo1 in the skeleton and muscular tissues, including bone, cartilage, tendons, and skeletal muscles. Here, we update the current understanding of the physiological and pathophysiological roles mediated by Piezo1 in the musculoskeletal system and discuss why Piezo1 should be regarded as a therapeutic target for musculoskeletal disorders, including osteoporosis (OP), OA, muscle atrophy, and intervertebral disc degeneration (IDD). Figure 1 provides an overview of the physiological functions of Piezo1 in the musculoskeletal system.

## Overview of Piezo1 channel

### Structure of Piezo1

Deciphering the structure of Piezo1 is crucial for gaining a comprehensive understanding of its function. Piezo1 is a large transmembrane protein without repetitive sequence and sequence homology to other known ion channels 32. Through cryo-electron microscopy techniques, it has been determined that Piezo1 consists of 2547 residues 33, and adopts a three-bladed, trimeric propeller-like architecture. This architecture features a central ion-conducting pore module topped with an extracellular cap domain 32. Each subunit of the Piezo1 structure contains a central ion-conducting pore and two peripheral modules 34. The central ion-conducting pore consists of outer helices, inner helices, intracellular C-terminal domains, and extracellular C-terminal domains (CEDs, which govern unitary conductance, ion permeability, and selectivity) 33. The peripheral mechanotransduction module includes a long beam-like structure and an anchor domain 8. The peripheral propeller blades house 38 transmembrane helices serve as the mechanosensing module 8. The detail of the structure and function of Piezo1 are described elsewhere 8. Overall, the trimeric structure of Piezo1 enables cooperative sensing of mechanical stimuli and subsequent activation of ion conductance. The precise arrangement and interactions of these domains enable Piezo1 to convert mechanical forces into electrical signals, contributing to its role in mechanotransduction.

### Pharmacological Modulators of Piezo1

Although mechanical stimulation plays a primary role in Piezo1 activity, it can also be regulated by pharmacology. Yoda1 was initially identified as a Piezo1-specific allosteric activator through high-throughput chemical library screening 35. It induces local conformational changes that result in the opening of Piezo1 pore, reducing the mechanical threshold required for channel activation 36. Yoda1 binds to the proximal end of the blade (residue 1961-2063) of Piezo1, enhancing membrane tension-induced blade motion through a wedge-like effect 36. The sensitivity of Yoda1 to protein mutations and structural modifications led to the development of analogues such as KC159, which contains 4-benzoic acid instead of the pyrazine moiety in Yoda1, and its potassium salt (KC289). These analogues have demonstrated equivalent or improved reliability, efficacy, and potency compared to Yoda1 in functional assays 37. Jedi1 and Jedi2 are alternative Piezo1 agonists that activate Piezo1 from the extracellular side of the blade 38. Yoda1 and Jedi1/2 exhibit synergistic effects, suggesting distinct activation mechanisms. Specifically, Yoda1 acts on the downstream beam, while Jedi1 and Jedi2 act on the upstream blade 38.

Several non-specific inhibitors of Piezo1 have been identified, including Grammostola spatulata mechanotoxin 4 (GsMTx4), ruthenium red (RR), and gadolinium (Gd^3+^). GsMTx4, a spider venom peptide, inhibits cation-sensitive mechanosensitive channels by reducing tension and lateral pressure on the membrane through insertion into the lipid bilayer 39. RR and Gd^3+^ are small molecules that can non-specifically block Piezo1 40. In addition to these non-specific blockers, Dooku-1 is a specific inhibitor that demonstrates inhibitory effects not only on Yoda1-induced activation of Piezo1 41, but also on the constitutively open Piezo1 channel 42. Certain Piezo1-interacting proteins, such as sarco/endoplasmic reticulum Ca^2+^-ATPase (SERCA) 36 and polycystin-2 (PC2) 43, can bind directly to Piezo1 and weaken the mechanosensitive current. Saturated and polyunsaturated fatty acids, such as arachidonic acid and eicosapentaenoic acid, can also inhibit Piezo1 activity in a non-specific manner 44.

### Expression and Distribution of Piezo1 in Musculoskeletal Tissues

The musculoskeletal system is vital for providing structural support, enabling movement, and maintaining overall homeostasis in the human body. Currently, there is significant research focus on understanding the role of Piezo1 in the musculoskeletal system. It is imperative to summarize the expression and distribution of Piezo1 based on current research findings. Within bone tissues, Piezo1 has been identified in various cell types residing in bone, including bone marrow mesenchymal stromal cells (BMSCs) 45, periosteal stem cells (PSCs) 46, osteoblasts 26, osteocytes 26, 47, 48, hypertrophic chondrocytes 26, and endothelial cells (ECs) 27. Piezo1 is predominantly expressed in differentiating osteoblasts and hypertrophic chondrocytes during limb development, suggesting its involvement in mechanotransduction during bone development 26. The expression of Piezo1 is upregulated in young mice after birth 26, but decreases with aging in cortical bone 49. In addition to aging, mechanical stimulation controls Piezo1 expression. Mechanical unloading has been observed to reduce Piezo1 expression 45, whereas mechanical stimulation, such as fluid shear stress (FSS) can increase its expression 50. In cartilage, transcriptomic analyses have revealed high expression of Piezo1 in both mice and human cartilage 51, 52. Following OA, Piezo1 is widely expressed in chondrocytes, as well as infrapatellar fat pad (IFP) and synovial membrane (SM) vessels 53. Within muscles, Piezo1 exhibits high expression in muscle stem cells (MuSCs), myotubes, and mature myofibers 23. Its expression increases at both mRNA and protein levels during myoblast differentiation 54. In addition, Piezo1 prefers to express in quiescent MuSCs, suggesting the role of Piezo1 in maintaining the MuSCs pool in skeletal muscles 23, 55. In tendon tissues, single-cell RNA sequencing revealed that *Piezo1* is mainly expressed in cells with high expression of *Mkx* and *Scx* genes, but relatively low expression in *Myod1^+^* Cluster 56. In intervertebral discs, Piezo1 is functionally expressed in nucleus pulposus (NP) and annulus fibrosus (AF) cells 57-59, and can be increased by mechanical stimulation 60. Understanding the expression patterns of Piezo1 in different musculoskeletal tissues provides valuable insights into its potential roles and mechanisms of action.

## Piezo1 in Musculoskeletal Physiology

The musculoskeletal system contains bone, cartilage, ligament, synovium, skeletal muscle, intervertebral disc, tendon, and the surrounding connective tissues 61. The relationship between the musculoskeletal system and mechanical stimulation is well established, particularly in relation to the Piezo1 channel 62-64. Here, we updated the current understanding of the physiological and pathophysiological roles mediated by Piezo1 in the musculoskeletal system. To date, different types of physical stimulations, such as hydrostatic pressure (HP) 65, FSS 66, Low-intensity ultrasound stimulation (LIPUS) 17, pulsed electromagnetic fields (PEMF) 67, and longitudinal substrate stretching 68 have been applied to elucidate the mechanosensitive processes in cells resident in the musculoskeletal system. We summarized the various physical stimulations on cell fate determination via Piezo1 activation in Table 1.

### Bone Formation and Remodeling

Bone is highly specialized and dynamic connective tissue, which is required for supporting muscles and providing the basis for mobility 69. Maintaining bone homeostasis relies on the delicate balance between osteoblast-mediated bone formation and osteoclast-mediated bone resorption 70. Mechanical loading is an essential regulatory factor in bone homeostasis 71. Cells within bone tissues, including MSCs 72, osteoblasts, osteoclasts, and osteocytes, process mechanosensory capabilities 73. These cells can sense mechanical stress through focal adhesion, plasma membrane receptors, and mechanosensitive ion channels 74. Recent investigations have highlighted the involvement of Piezo1 in bone development and mechanosensing for bone formation. Conditional deletion of *Piezo1* in specific populations, such as osteoblasts and osteocytes, leads to compromised bone structure and reduced strength 75, consequently resulting in developmental bone defects and an increased susceptibility to bone fractures 26. Notably, these detrimental outcomes are proposed to be related to mechanical loading, given that the low bone mass phenotype in *Piezo1^Prrx1^* mice only occurs in load-bearing bones 76. Here, we summarized the role of Piezo1 in different cell populations resident in bone tissues.

#### Piezo1 resident in different cells regulates bone development and affects bone formation

##### Bone marrow mesenchymal stromal cells (BMSCs)

Deletion of *Piezo1* in MSCs disrupts osteoblast differentiation and promotes bone resorption, resulting in the occurrence of spontaneous bone fractures 26, 77, 78. Conversely, Piezo1 activation by Yoda1 enhances the proliferation and osteogenic differentiation capability of Gli1^+^ BMSCs *in vitro* and *in vivo* 45. Mechanosensitive ion channels, including TRPV4, Piezo1, and Piezo2, have been implicated in mediating the osteogenic differentiation of BMSCs in response to mechanical stimulation 77. Yoda1 has been shown to induce a TRPV4-dependent Ca^2+^ response by activating Piezo1 79. However, the specific interplay and functional relationship between Piezo channels and TRPV4 in the context of osteogenic differentiation remains to be explored. Future research is warranted to elucidate the precise mechanisms and signaling pathways involved in the crosstalk between TRPV4 and Piezo channels during osteogenic differentiation of BMSCs. Notably, a study has demonstrated that the C-terminus of Piezo1 is crucial for the mechano-transduction of osteoblastic differentiation in BMSCs via the ERK1/2 signaling pathway 80. Under unloading conditions, Piezo1 activation by Yoda1 mitigates bone loss through the Wnt/β-Catenin signaling pathway 45. Besides, Piezo1 expression has been identified in Oln^+^ BMSCs 75, 81. In the central bone marrow and near the endosteum of diaphyseal bone, a peri-arteriolar niche that harbors leptin receptor (LEPR) and osteolectin (Oln) double-positive stromal cells was identified by Shen *et al.* 82. These LEPR^+^Oln^+^ BMSCs exhibit high proliferation rates and preferentially differentiate towards osteogenesis and lymphopoiesis through Piezo1 signaling. In addition, the number of LEPR^+^Oln^+^ BMSCs decreases with aging, suggesting their involvement in age-related bone loss. Deletion of *Piezo1* in LEPR^+^Oln^+^ BMSCs leads to reduced bone mineral density and cortical bone thickness. In contrast, mechanical and pharmacological stimulation of Piezo1 with Yoda1 on LEPR^+^Oln^+^ BMSCs promotes bone formation and supports bacterial clearance following bone fracture 82.

##### Periosteal stem cells (PSCs)

PSCs resident in the periosteum are vital for bone fracture healing 83. Single-cell RNA sequencing data showed that PSCs are the ancestors of osteoblasts 46. Piezo1 is upregulated after bone fracture 46. Yoda1 treatment directly enhances migration and osteogenic differentiation of PSCs, indirectly promoting angiogenesis *in vitro* and* in vivo*. Yap/β-catenin pathway is another downstream effector of the Piezo1 channel 46.

##### Osteoblasts

Piezo1 is essential for osteoblast proliferation, migration, and differentiation 26, 75, 79. Conditional deletion of *Piezo1* in osteoblast, using mouse models such as *Piezo1^OcnCre^*, *Piezo1^Dmp1Cre^*, *Piezo1^Sp7Cre^* mice, results in impaired bone formation and reduced bone mass 26, 81. Specifically, deletion of *Piezo1* in Prrx1^+^ cells (*Piezo1^Prrx1Cre^*) results in multiple bone fracture, shortened long bones, and these effects are further exacerbated in *Piezo1/2* double knockout mice. Of note, *Piezo2* conditional knockout mice (*Piezo2^Prrx1Cre^*) undergo normal skeletal development, indicating that Piezo1 plays a major role in MSCs during bone development 26. The authors also generate *Piezo1^Sp7Cre^* and *Piezo2^Sp7Cre^* mice to demonstrate that Piezo1/2 are essential for bone mass maintenance through mechanical stimulation 26. Bulk RNA Sequencing and further experiments revealed that Piezo1/2 regulate osteoblast differentiation by modulating Wnt/Ctnnb1 and Yap1 pathways 26. Apart from intramembranous ossification, Piezo1 also has a key function in the early stages of osteoblast differentiation, affecting bone formation through endochondral ossification 78. *Piezo1^Runx2Cre^* mice exhibit reduced cortical thickness and increased cortical porosity, with a phenotype that is more severe and distinct from that of *Piezo1^Dmp1Cre^* mice. Osteoblasts isolated from *Piezo1^Runx2Cre^* display an unusual flattened appearance, increased chondrogenic differentiation potential, and reduced osteogenic differentiation ability 78.

The effects of mechanical loading on bone formation are partly attributed to the promotion of Piezo1 signaling in osteoblasts 75, 81.* In vitro* study revealed that FSS exposure for 1 hour increases *Runx-2* gene expression in MC3T3-E1 osteoblasts, and the increased *Runx-2* gene expression is eliminated by *Piezo1* gene deletion 47. Piezo1 activates the Akt/GSK-3β/β-catenin pathway following the phosphorylation of Akt and phosphorylation of GSK-3β, which is recognized as a partial mechanism 47, and it induces NFAT/YAP1/ß-catenin complex formation by stimulating Calcineurin 26. Apart from FSS, LIPUS induces Erk1/2 phosphorylation and perinuclear F-actin polymerization in a Piezo1-dependent manner to promote MC3T3-E1 cell migration and proliferation 17. Also, Piezo1 regulates the phosphorylation of Erk1/2 and p38, and enhances BMP2 expression through HP or Yoda1 treatment 26. Dentin matrix protein 1 (DMP1), an extracellular matrix protein belonging to the small integrin-binding ligand N-linked glycoprotein (SIBLING) family, is crucial for bone mineralization 84. Mechanical loading, such as body weight-bearing, increases the production of kinase FAM20C in osteoblasts, promoting DMP1 secretion via activating Piezo1 channel 85. The secreted DMP1 can convert type H vessels into type L, inhibiting bone growth and promoting bone mineralization by impeding VEGF signaling. However, Piezo1 in the gut has negative effects on osteoblast activity. Conditional deletion of *Piezo1* in the gut increases bone mass accompanied by decreased serum 5-HT levels 21. Sugisawa *et al.* generated *LysM*-*Piezo1^flox/flox^* mice and *Col1a1*-*Piezo1^flox/flox^* mice, and found no significant change of bone volume and serum bone markers after Piezo1 deletion in myeloid and osteoblast 21. They hold the view that Piezo1 in myeloid and osteoblast is not that essential for bone metabolism 21. Such discrepancy warrants further study to reach a consensus.

##### Osteoclasts

Conditional knock-out *Piezo1* in osteoclast (*Piezo1^CtskCre^*) exhibited normal body weight, bone mass, and bone resorption process 76. However, multiple evidence showed that Piezo1 in osteoblasts responds to mechanical stimulation to maintain bone size and mass via regulating osteoblast-osteoclast crosstalk 75, 76.

##### Osteocytes

Osteocytes, the most abundant cell type in bone tissue, play crucial roles in sensing and transducing mechanical stimulation and regulating bone formation and remodeling 86. To investigate the role of Piezo1 in osteoblasts and osteocytes, *Piezo1^Dmp1Cre^* mice were generated. These mice exhibited low bone mineral density, decreased cortical bone thickness, and a diminished response to mechanical loading. The authors identified that Wnt1, Yap1, and TAZ are the downstream effectors of Piezo1 75.

Piezo1 is co-localized with Connexin43 hemichannels (Cx43 HCs), which facilitates the exchange of small molecules in the extracellular environment on the surface of osteocytes 50. FSS increases the expression of Piezo1 and enhances the co-localization of Piezo1 and Cx43 HCs 50. Piezo1 activation by Yoda1 increases intracellular Ca^2+^, which opens Cx43 HCs and Panx1 channels through activating the PI3K-Akt signaling pathway. Additionally, these activated channels promote ATP release, which in turn activates P2X receptors and sustains intracellular Ca^2+^ signaling 50. In MLOY4 osteocytes, Piezo1-mediated FSS enhances Osteoprotegerin (OPG) expression and reduces nuclear factor-Kappa-B Ligand (RANKL) expression through NOTCH3 87. Knockdown of *Piezo1* in osteocytes reduces osteogenic makers in osteoblasts, even when exposed to LIPUS 88.

Aging is a natural and inevitable process that occurs in living organisms. It is associated with an increased risk of developing various age-related health conditions and diseases. Loss of bone mass occurs in the aging skeleton, often characterized by osteoporosis and an increased risk of fracture 89. *Piezo1^Dmp1Cre^* mice with aging exhibited enhanced endocortical expansion, cortical porosity, and increased osteoclast formation through elevating *Tnfrsf11b* expression 49. Sclerostin, highly expressed in osteocytes, is a bone formation inhibitor and one of the molecular regulators in bone homeostasis 90. Mechanical stretch increases the phosphorylation of Akt and then reduces *Sost* expression through Piezo1 activation 48. However, increased *Sost* (gene of Sclerostin) expression was observed in *Piezo1^OcnCre^* mice, which indicates that osteocytes may coordinate with osteoblasts in bone homeostasis 81.

##### Chondrocytes

The involvement of chondrocytes in endochondral ossification is closely linked to bone formation. Conditional knockout *Piezo1* in chondrocytes (*Piezo1^Col2a1Cre^*) reduces trabecular bone formation, suggesting that the presence of Piezo1 in growth plate chondrocytes is responsible for trabecular bone formation 78. Endochondral ossification is one of the most essential mechanisms involved in ankylosis progression in ankylosing spondylitis (AS). Ablation of *Piezo1* in chondrocytes (*Piezo1^Col2a1Cre^*) can inhibit pathological new bone volume and alleviate the AS phenotype. CaMKII activation is the downstream pathway of Piezo1-mediated pathological new bone formation in AS 91.

##### Endothelial cells (ECs)

Piezo1 is essential for local vascular growth. Global knockout of *Piezo1* in mice leads to fetal lethality due to obvious deformity during vascular development 92. Piezo1 in ECs induces angiogenesis, thereby promoting bone fracture healing 27. *Piezo1^Cdh5Cre^* impedes bone fracture healing by altering osteoblastic activity in the early stages and reducing bone remodeling in the late stages 27. In addition, both Piezo1 and Piezo2 are expressed in gut epithelial cells 93, 94, and both are involved in the production of serotonin (5-HT) 21, 95. 5-HT produced by the gut is a negative regulator of bone metabolism 96. It has been reported that conditional deletion of Piezo1 in intestinal epithelium leads to increased bone mass 21. This phenomenon can be attributed to impaired 5-HT production in the gut 21, highlighting the relationship between 5-HT production and bone metabolism.

##### Myeloid-lineage cells (MCs)

The periosteum is responsible for cortical bone development and strain-adaptive remodeling 97. It consists of nerves, blood vessels, and multiple types of cells resident in the periosteum, including periosteal progenitors and myeloid-lineage cells, which collectively create a pro-osteogenic microenvironment 98. Among them, macrophages are crucial for bone remodeling and regeneration. Deng *et al.* have identified a specific subtype of macrophages, CD68^+^F4/80^+^ macrophages, that regulate bone remodeling in response to mechanical stimulation. Specifically, compression increases the number of CD68^+^F4/80^-^ MCs and promotes their differentiation into CD68^+^F4/80^+^ macrophages by upregulating Piezo1 expression. CD68^+^F4/80^+^ macrophages secret latent TGF-β1, and Thbs1, which activate TGF-β1, consequently mobilizing and recruiting more osteoprogenitor cells to the periosteal bone surface, thereby promoting bone regeneration under mechanical stimulation 99. The role of macrophages in osteogenesis is further supported by a recent study conducted by Cai *et al.* 100. They identified that mechanical stretch enhances M2 macrophage polarization and secretion of TGF-β1 through the Piezo1 channel to promote the proliferation, migration, and osteogenic differentiation of BMSCs 100.

**Figure 2:**
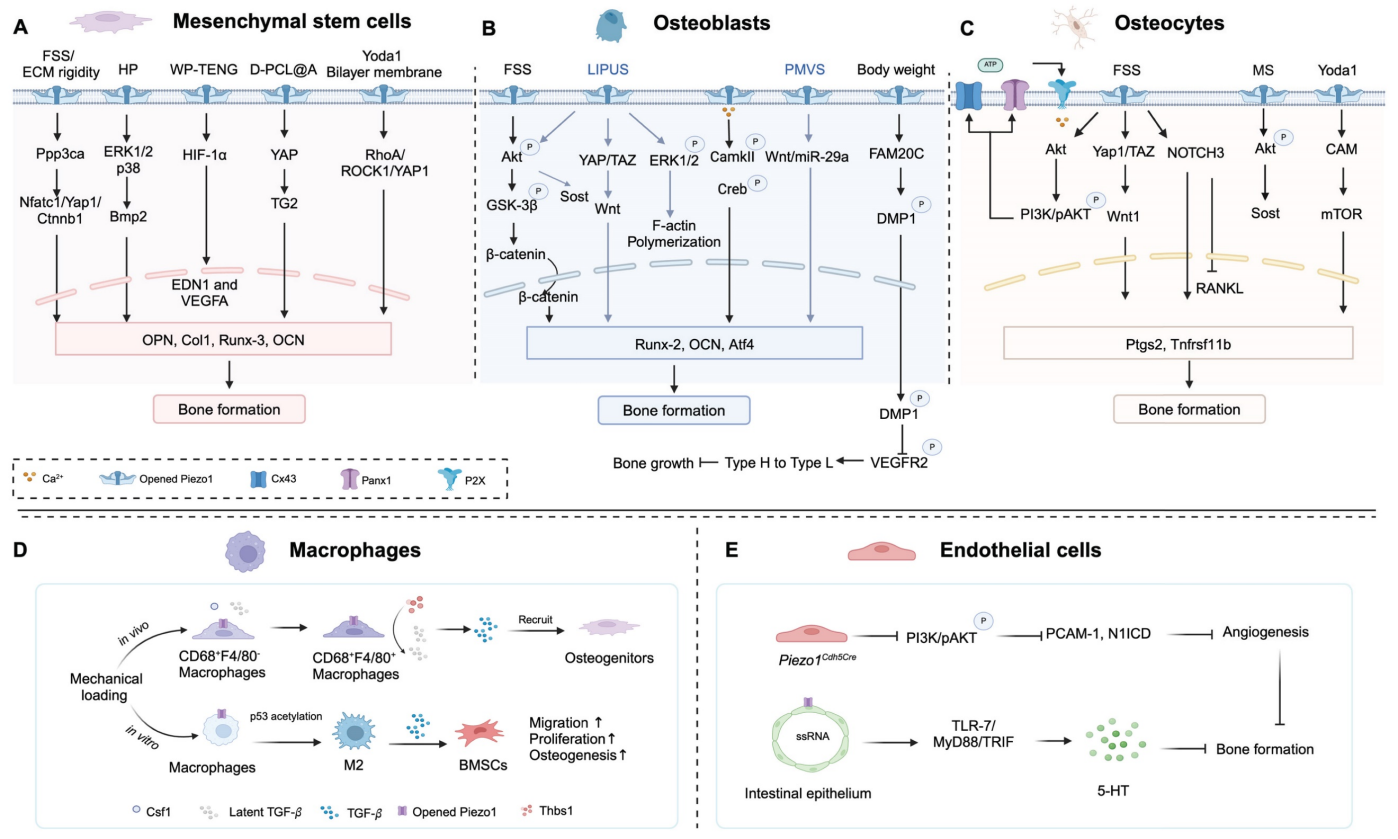
** Piezo1 located in different cells regulates bone formation.** (A) Mechanical stimulation by fluid shear stress (FSS), ECM rigidity, hydrostatic pressure (HP), and biomaterials (WP-TENG, D-PCL@A, and Yoda1 Bilayer membrane) activates Piezo1 channels in mesenchymal stem cells (MSCs), further activates downstream signaling pathway. (B) Activation of Piezo1 in osteoblasts (MSC-derived osteoblasts and MC3TC-E1) by FSS, LIPUS, Piezoelectric micro-vibration stimulation (PMVS), and increasing body weight, and the downstream signaling pathway. (C) FSS, mechanical stretch (MS), and Yoda1 activate Piezo1 in osteocytes to enhance bone formation. (D) Piezo1 mediates mechanical loading enhances M2 macrophage polarization and promotes proliferation, migration, and osteogenic differentiation of BMSCs via secreting TGF-β1. (E) Conditional knockout Piezo1 in endothelial cells (*Piezo1^Cdh5Cre^*) impedes bone formation, while Piezo1 activation in intestinal epithelium inhibits bone formation mediated by 5-HT production. Created with BioRender.com.

Taken together, Piezo1, in response to mechanical loading, is strategically situated within a multitude of cells, such as bone cells, chondrocytes, macrophages, and endothelial cells, exerting an impact on bone homeostasis. In this regard, Piezo1 as a key mechanosensor for bone formation, may be a novel therapeutic target for OP treatment. The roles of Piezo1 in different cell types and the related signaling pathways are illustrated in Figure 2.

### Cartilage Homeostasis

**Figure 3:**
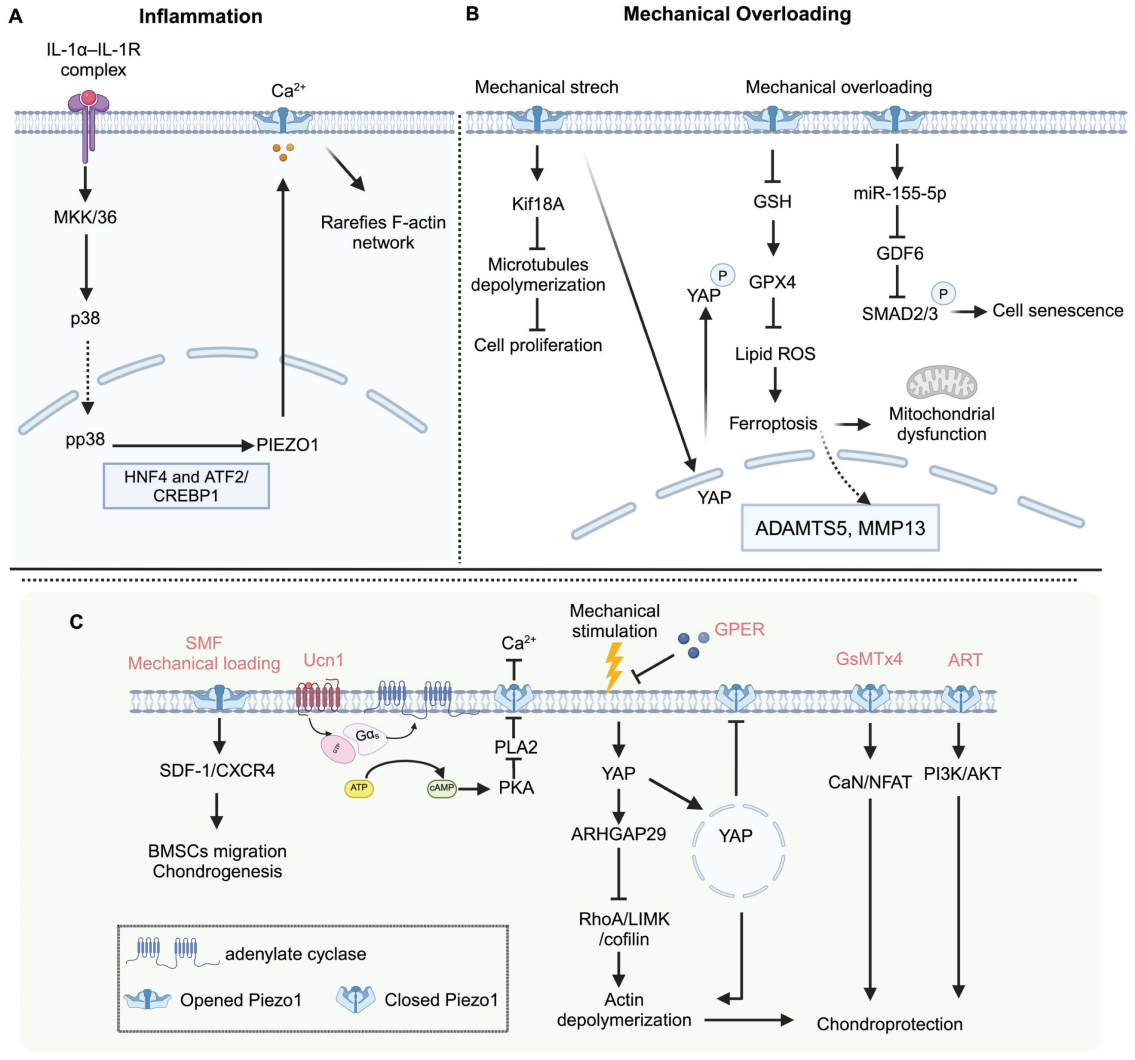
**Piezo1 mediated chondrocytes mechanotransduction and potential therapeutic targets for OA.** (A) Inflammation cues increase the mechanosensitivity of chondrocytes mediated by Piezo1 to mechanical loading. (B) Mechanical overloading induces chondrocyte ferroptosis in OA via Piezo1 activation (C) Static magnetic field (SMF), and appropriate mechanical loading promotes BMSCs chondrogenic differentiation via Piezo1 activation. Urocortin-1 (Ucn1), G protein-coupled estrogen receptor (GPER), GsMTx4 (a peptide of Piezo1 inhibitor), and Artemisinin (ART) protect chondrocytes from damage to alleviate OA symptoms. Created with BioRender.com.

Articular cartilage is a thin layer of specialized connective tissue that provides a smooth surface to minimize friction and transmits mechanical loading to the subchondral bone 101. Chondrocytes, the cells within cartilage, sense and respond to mechanical stress to maintain cartilage homeostasis 102. Excessive mechanical loading can cause changes in chondrocyte volume and deformations 103. Piezo channels communicate with each other and even other ion channels, like the TRPV4 channel 104-106. Both Piezo1 and TRPV4 are active in chondrocytes. They show similar responses to nanoscale deflection-stimuli in chondrocytes, and the function deficiency of each ion channel can be compensated by the other 105. TRPV4 mainly senses physiologic stimulation in cartilage, and Piezo channels mediate excessive mechanical loading 107. Mechanical loading influences chondrocyte death 106, 108 and recruits more stem cells, contributing to cartilage repair via the Piezo-mediated pathway 109. Nevertheless, excessive mechanical loading induces Ca^2+^ influx with increased Piezo1 level, resulting in chondrocytes apoptosis 110 and senescence 111. GsMTx4, an effective Piezo1 inhibitor, can enhance chondrogenic markers 78, increase cartilage matrix production, inhibit chondrocytes apoptosis, and protect articular cartilage from mechanical injury through the calcineurin (CaN)/ nuclear factor of activated T cells 1 (NFAT1) signaling axis 106, 110. However, GsMTx4 exhibits non-specificity towards Piezo1, indicating that the observed effects may not be mediated solely by this channel. In addition, Piezo1 activation induced by extreme mechanical stimulation and Yoda1 accelerates chondrocyte senescence 112, 113, and Yoda1 reduces *Col10a1* gene expression in ATDC5 chondrogenic cells 78. Urocortin (Ucn1), a 40 amino acid long peptide, has an antiresorptive effect in bone tissue 114. Recently, a new pathway of Ucn1 that promotes chondrocyte survival has been identified. Ucn1 protects chondrocytes through maintaining Piezo1 in a closed conformation mediated by the corticotropin-releasing factor receptor 1 (CRF-R1 receptor) of Ucn1 115, 116. The above evidence indicates that antagonism of Piezo1 may be a promising therapeutic approach for OA patients. Figure 3. shows the role of Piezo1 in cartilage metabolism.

### Skeletal Muscle

Skeletal muscle accounts for approximately 40% of total body mass 117, and plays a vital role in facilitating movement, providing stability, maintaining posture, and orchestrating various essential physiological processes in the body 118. Skeletal muscle with high plasticity and exceptional regeneration capacity is largely affected by mechanical stimulation 119. Mechanosensitive ion channels are key players in skeletal muscle homeostasis. They can enhance the cytoskeleton and prevent cell lysis by sensing excessive loading on the sarcolemma 120. Among them, Piezo1 is a crucial mechanosensitive ion channel expressed stably in satellite cells and is responsible for myotube formation 54.

#### Piezo1 controls myotube formation through regulating muscle stem cell (MuSCs) fate

Myotubes formed by myoblast fusion are one of the essential steps of skeletal muscle development 121. This process of myoblast fusion requires membrane remodeling and mechanical forces 54. Various cellular events take place during myoblast fusion, such as cell-cell communication, elongation, adhesion, and alignment of myoblast membranes 121. Within skeletal muscle tissue, ion channels play a pivotal role in muscle growth. Piezo1, as one of the mechanosensitive ion channels, regulates myotube formation and cortical actomyosin assembly through controlling the influx of Ca^2+^ across the cell membrane 28, 122. In a study by Ortuste and colleagues, treatment of myotubes with Yoda1 (at concentrations of 30 and 100 µM) for 1 minute significantly induced cell fusion, whereas a 30-minute treatment with 100 µM of Yoda1 reduced cell fusion 54. Knockdown of *Piezo1* in myoblast reduces myoblast fusion and myomaker expression, thereby impeding myotube formation 54. In *Piezo1*-deficient C2C12 cell lines, abnormal morphology is observed during myotube formation, characterized by excessive cell fusion and defects in cell elongation 28. Phosphatidylserine (PS), a phospholipid with a negative charge, is typically located in the inner leaflet of the plasma membrane 123. During myotube formation, the inner leaflet PS moves to the outer leaflet and then can be recognized by PS receptors to facilitate fusion with adjacent myoblasts 124, 125. The tempo-spatial activation of Peizo1 is regulated by PS flippase-mediated translocation, consequently influencing myoblast fusion and elongation 28.

Muscle stem cells (MuSCs) play a critical role in muscle growth and regeneration, both in physiological and pathological states 126. These cells respond promptly to exercise and injury, undergoing activation, proliferation, and differentiation into myoblasts. A small fraction of MuSCs retain their quiescent state within the MuSCs pool through self-renewal 127. Currently, little is known about how the surrounding environment influences the transition of MuSCs between quiescence and activation. Aging populations often exhibit muscle weakness and reduced muscle regeneration 128. Piezo1 is indispensable for MuSCs proliferation, differentiation, and even alleviation of cellular senescence 55. To investigate the impact of Piezo1 in MuSCs on muscle injury, a conditional knockout strain of *Piezo1* in MuSCs (*Piezo1^Pax7Cre^*) was generated. In *Piezo1^Pax7Cre^* mice, elevated levels of P53, increased ROS formation, and decreased muscle regeneration ability were observed 55. It is suggested that cPKC activation, mediated by increased Ca^2+^ influx through T-Type Ca^2+^ channels, plays a key role in this process 55. This is consistent with the notion that the concentration of Ca^2+^ is associated with cellular senescence in muscle fibers 129. The administration of Pifithrin-α (PFT-α), a P53 inhibitor, reduced the rate of MuSCs senescence, ultimately improving muscle regeneration. Piezo1 is involved in the regulation of P53 expression and ROS production, thereby contributing to the maintenance of the MuSCs pool by inhibiting MuSCs senescence.

**Figure 4:**
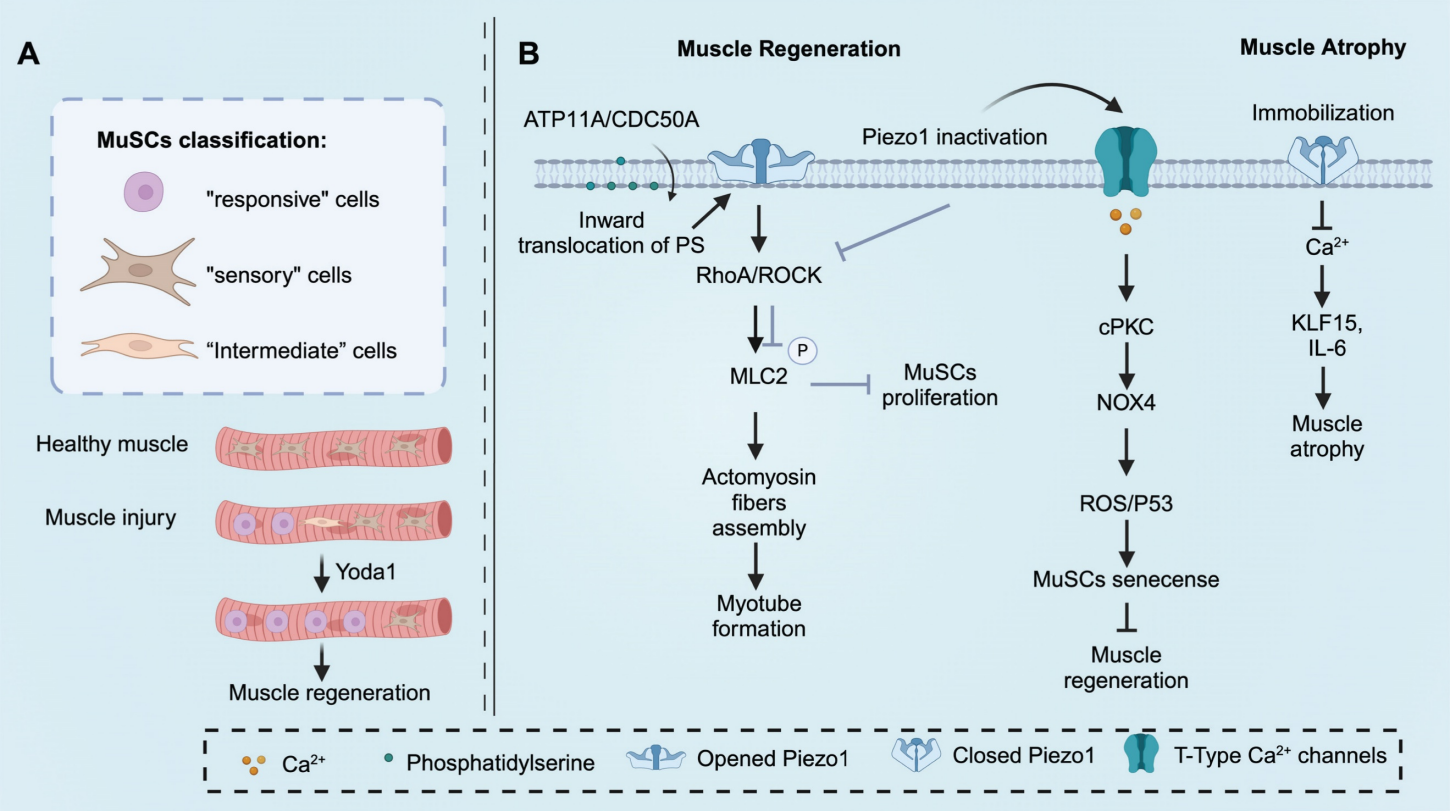
**Piezo1 functions in skeletal muscle.** (A) Piezo1 orchestrates muscle satellite cells (MuSCs) morphological states after muscle injury, which is essential for muscle regeneration and maintenance. Pharmacologically activate Piezo1 by Yoda1 prime MuSCs toward more “responsive” cells. (B) The inward translocation of phosphatidylserine is the precondition of Piezo1 activation. Piezo1 activation enhances myotube formation, MuSCs proliferation, and inhibits MuSCs senescence to promote muscle regeneration. In addition, Piezo1 downregulation results in muscle atrophy. Created with BioRender.com.

The morphology of MuSCs also plays a crucial role in determining their functions 130. Isolated MuSCs with few protrusions are considered relatively fragile and more susceptible to the influence of the surrounding environment 131. Recent studies have demonstrated that MuSCs with fewer protrusions exhibit an initial response after injury, followed by MuSCs with more protrusions, indicating their rapid responsiveness 130. The authors categorized quiescent MuSCs into three subtypes, “responsive” cells (small and round cells with zero or one protrusion), “intermediate” cells (middle-size cells with two or three protrusions), and “sensory” cells (large and less rounded cells with four or more protrusions). Pharmacological activation of Piezo1 by Yoda1 has been shown to promote a shift of MuSCs towards the “responsive” cell subtype in Pax7-EGFP mice 130. To investigate the role of Piezo1 in the functional transition between these subtypes, *Piezo1^Pax7Cre^* mice were utilized. After muscle injury, *Piezo1^Pax7Cre^* mice exhibited an increase in "intermediate" cells and a decrease in "responsive" cells 130. These findings suggest that Piezo1 is essential for maintaining the morphology of MuSCs, and downregulation of Piezo1 may lead to reduced proliferation of MuSCs, thereby disrupting muscle homeostasis and regeneration 130. The function of Piezo1 in skeletal muscle is shown in Figure 4.

### Tendon

Tendons are mechanosensitive soft tissues that connect muscle to bone to enable ambulation and suffer high mechanical loading transmitted by muscles 132. Mechanical loading is essential for tendon homeostasis in humans and animal models 133, and it can have dual effects on the tendon healing process 134. Mechanical overloading is a well-known extrinsic factor that results in tendon injury 135. However, the prolonged unloading process also exerts detrimental effects on tendon mechanical properties 136. Besides, under aberrant mechanical stimulation, excessive biological factors, like prostaglandins, metalloproteinases, and some growth factors can be produced 137, and the differentiation capabilities of tendon stem cells are altered 138. However, the underlying mechanism of how resident tendon cells respond to mechanical forces and translate into biological signals remains unknown.

#### Piezo1 activation enhances tendon stiffness

Currently, only two research groups have published research on the role of Piezo1 in tendons. Piezo1 is essential for tendon function 24, 56. Both loss-of-function mice and gain-of-function mice have been utilized to investigate its effects. Passini and his colleagues developed two devices, a tensile stretching device, and a microfluidics flow chamber to examine the Ca^2+^ influx after mechanical stimulation *ex vivo*. Mechanical forces were found to trigger Ca^2+^ influx in tenocytes 24. Similarly, Nakamichi *et al.* observed an accelerated Ca^2+^ influx after the specific agonist Yoda1 treatment, consistent with the findings of Passini *et al.* 56. Knocking out several general ion channels by CRISPR-Cas9 genome editing technique revealed that only cells with *Piezo1* depletion exhibited a decreased response to shear stress 24, suggesting that the Piezo1 channel may be the primary sensor of shear force stimulation. To further identify the crucial role of Piezo1 in responding to mechanical loading, Passini *et al*. generated the conditional depletion of *Piezo1* in tenocytes (*Piezo1^ScxCre^*) mice. The results showed reduced tendon stiffness in *Piezo1^ScxCre^* mice 24, which could be increased over two weeks of Yoda1 treatment.

Over 25 gene mutations in *Piezo1* are associated with human diseases 139. E756del, a common Piezo1 Allele, is prevalent in one out of three African populations, including African Americans and Jamaicans 140. Emerging evidence showed that the E756del mutation regulated human physical performance. One double-blinded trial investigated the E756del gene in healthy African Americans 24. The step motion, such as countermovement jump, is often a training process in athletics 141. The human E756del carriers performed better in the drop countermovement jump test, which could induce high degrees of tendon loading 24. Another research group found an increased frequency of E756del in Jamaican sprinters compared with controls from Jamaica 56. These data indicated that the Piezo1 E756del is involved in higher physical performance. To mimic the gain-of-function variant E756del in humans, the R2482H mutation of *Piezo1* was introduced in mice 140. Passini *et al.* observed that R2482H *Piezo1* mice had elevated stiffness and stronger plantaris tendons due to the denser collagen cross-link network 24. Nakamichi* et al.* also found that the width and the cross-section of collagen fibrils of Achilles tendon R2482H *Piezo1* mice were 1.2-fold wider than WT mice 56. *Piezo1^GOF^* mice performed better jumping and high-speed running abilities 56. However, this phenotype was not observed in muscle-specific Piezo1 gain-of-function mice 56, the contraction of muscles transmits the kinetic energy to the joints by tendons and ligaments 56. So, it is reasonable to speculate that damaged tendons induce secondary changes in skeletal muscle tissues. In addition, RNA sequencing showed upregulation of tendon-related genes (Mkx and Scx), collagen matrix, and non-collagen matrix genes in R2482H Piezo1 mice 56. Also, Piezo1 enhances Mkx and Scx, promoting tendon synthesis by inducing the nuclear translocation of multiple NFATCs (NFATC1, NFATC2, NFATC3, and NFATC4). Therefore, controlling Piezo1 activation may hold promise for improving tendon function in tendon-related diseases such as tendon rupture and tendinopathy.

### Intervertebral Disc

#### Piezo1 activation increases NP and AF cell apoptosis and senescence

**Figure 5:**
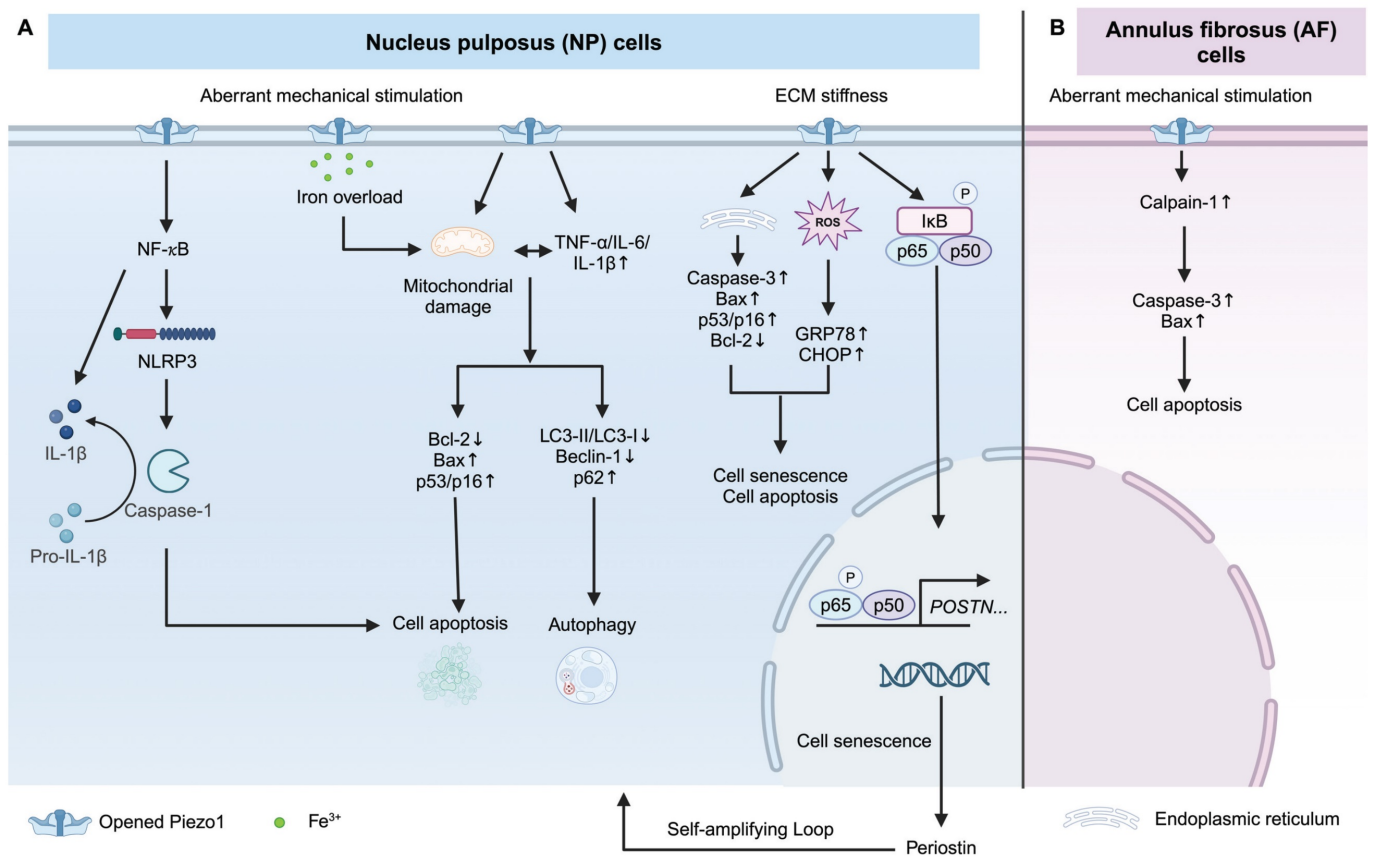
**Piezo1 mediates apoptosis and senescence of NP and AF cells.** (A) Piezo1 activation under excessive mechanical stimulation and stiff matrix triggers apoptosis and senescence of NP cells. (B) Excessive mechanical stimulation induces AF cell apoptosis via Piezo1 activation. Created with BioRender.com.

Intervertebral disc (IVD) consists of NP in the center, surrounding AF, and cartilaginous endplate (CEP), which is linked to superior and inferior vertebral bodies. Piezo1 mediates inflammation by activating Nod-like receptor protein 3 (NLRP3) inflammasome 57, 142. Mechanical stretch on NP cells induces IL-1β production via Piezo1-mediated NLRP3 inflammasome activation 57, with the downstream effector being the NF-κB pathway 57, 59. Excessive mechanical stress leads to senescence and apoptosis of human NP cells via Piezo1 overexpression and secretes some pro-inflammatory factors (such as TNF-α, IL-6, and IL-1β), resulting in ECM reduction and autophagy inhibition 60. Knockdown of *Piezo1* protects NP cells from apoptosis by reducing the ratio of mitochondrial membrane potential turnover induced by aberrant mechanical stimulation 143. In addition, the stiffness of the ECM during IDD contributes to the activation of Piezo1 144. A stiff matrix (25 kPa) activated Piezo1, leading to endoplasmic reticulum (ER) stress and oxidative stress, thereby inducing senescence and apoptosis of human NP cells 25. It was observed that a stiff matrix increases the secretion of periostin from human NP cells, which in turn activates the NF-κB pathway. This activation further enhances periostin expression, accelerating the senescence of NP cells 59. This self-amplifying loop between periostin and NF-kB can be triggered by Piezo1 activation, resulting in IDD 59. Besides, Ke *et al.* also identified that Piezo1 activation by matrix stiffness promotes NP cell apoptosis via activating the ERK1/2 pathway 145. Piezo1-mediated iron overload disrupts iron metabolism and exacerbates ferroptosis in NP cells. Importantly, Piezo1-induced iron influx is independent of the transferrin receptor (TFRC), a well-recognized iron gatekeeper 146. In AF cells, excessive mechanical loading promotes AF cell senescence via the Piezo1/Ca^2+^/Calpain2/Caspase3 pathway 58. Overall, Piezo1 activation enhances inflammatory response and promotes NP and AF cells senescence, and apoptosis. Piezo1 may be a potential therapeutic target for IDD treatment. How Piezo1 regulates NP and AF cell fate is shown in Figure 5.

## Piezo1 in the Physical Performance

One of the benefits of exercise comes from the increased blood flow 147. Currently, the idea about how blood flow sensing during exercise is contradictory 148, 149. Baroreflex is an essential part of regulating blood pressure. Baroreceptor nerve endings can sense increased blood pressure, thus transferring the afferent signals to the central nervous system to redistribute blood flow 150. Piezo1 works as the baroreceptor mechanosensor for blood pressure regulation. Piezo1 is expressed in nodose and petrosal sensory ganglia, where baroreceptor cell bodies are located 151. Conditional deletion of *Piezo1* in nodose and petrosal sensory ganglia results in disturbing baroreflex and aortic depressor nerve activity 151. However, the role of Piezo1 in regulating blood pressure is not restricted to controlling the function of the baroreceptor reflex.

Endothelial cells lie between blood flow and the vascular wall and are essential for physiology and pathology. These cells experience hemodynamic forces, especially shear stress caused by fluid flow 152. Identifying the molecular sensor will enhance our understanding of the benefits of exercise. Piezo1 channel, highly expressed in endothelial cells 92, is crucial for vascular development. Deletion of endothelial *Piezo1* leads to embryonic lethality 92. Piezo1 is a key shear stress sensor of elevated blood pressure during physical activity 153. Physical activity-induced increases in blood pressure were mitigated in *Piezo1^Cdh5Cre^* mice, whereas no significant blood pressure elevations were observed during periods of physical inactivity 153. Piezo1 controls the vascular tone and blood pressure by regulating flow-induced ATP release and initiating the downstream signaling pathways 13. Besides, the lack of Piezo1 in endothelial cells reduces running wheel performance and causes weight loss in mice, indicating that Piezo1 regulates physical performance via redistributing blood flow 153. Recently, Wang* et al.* found a novel Piezo1 regulator, cartilage oligomeric matrix protein (COMP). They identified that COMP increased Ca^2+^ influx, eNOS activity, and nitric oxide production to regulate blood pressure 154. In addition, microvascular density is essential in cardiovascular function and maintaining normal physical performance 155. *Piezo1^Cdh5Cre^* mice showed lower physical activity without altering the desire for exercise 156, owing to microvascular rarefaction in muscle mediated by endothelial cell apoptosis 156.

Nitric oxide (NO), one of the vasoactive mediators triggered by FSS, is an essential endothelial vasodilator factor for controlling vascular tone and blood pressure 157. *Piezo1* deficiency in endothelium impedes NO formation and vasodilation in response to flow, leading to the development of hypertension 13. Utilizing titanium dioxide-trapping combined with mass spectrometry, it has been revealed that the deletion of *Piezo1* affects endothelial nitric oxide synthase (eNOS) under both static and shear-stress conditions 92. Also, increased shear stress by elevated blood flow during physical exercise activates the Piezo1 channel, thereby maintaining the microvascular structure by regulating eNOS/TSP2 paracrine signaling to stabilize muscle function 156.

The excellent physical performance relies on strong tendons 158. The tendon stiffness directly affects the muscle power outputs 159. Notably, Piezo1 gain-of-function in tendons, resulting in increased stiffness, has been found to enhance the physical performance in both humans and mice 24, 56. Intriguingly, Piezo1 senses physical activity to redistribute blood flow, which is closely associated with physical activity 153. Piezo1 activation mimics the effects of physical exercise. Beech *et al.* defined Piezo1 as an “exercise sensor”, and the specific activator, Yoda1, was named as “exercise pill” 160. In clinical scenarios, appropriate physical activity is beneficial for patients with musculoskeletal disorders, including OP 161, OA 162, muscle atrophy 163, IDD 164, and tendinopathy 165. In future work, this “exercise pill” may be applied to mimic the benefits of exercise-based physical therapy to treat bone, cartilage, muscle, and tendon-related pathologies in the rehabilitation phase.

## Implications of Piezo1 in Musculoskeletal Disorders

### Osteoporosis and Bone Health

OP is an aging-related disease with low bone mineral density (BMD), which leads to bone fragility and prone to fractures. With the increasing age and a higher prevalence among females, it becomes an increasing burden on health care worldwide 166. The incidence of OP is approximately 13% in China 167. In the United States, 10 million people aged over 50 were diagnosed with OP 168. Piezo1 expression is declined in OP patients 81. Conditional deletion of *Piezo1* in osteoblasts and chondrocytes results in severe OP 76, 78. However, Piezo1 activation can attenuate bone loss under the conditions of unloading, OVX, and aged mice models 45. Interestingly, genetic variants on Piezo1 are associated with human low BMD and fracture. 14 Top overlapped Piezo1 single nucleotide polymorphisms (SNPs) were found to be related to low BMD. Among them, SNPs rs62048221 from calcaneus significantly affects BMD by regulating Piezo1 expression and the activity of cis-regulatory elements 169. A bioinformatic analysis of the DEGs affected by the Piezo1 in different bone tissues and cells showed that Piezo1 gene is associated with mineral absorption 170. Marouli *et al.* have also identified the relationship between Piezo1 SNPs and human body height reduction 171. Furthermore, the lower Piezo1 expression contributes to bone aging 26. Apart from disuse osteoporosis, Piezo1 activation can also reduce bone loss in OVX and aging mice models 45.

Numerous biomaterials have been developed to enhance bone regeneration. Shi *et al.* conducted a study where they modified polycaprolactone (PCL) surfaces using 3,4-Dihydroxyphenylalanine (DOPA) and alendronate (AL). They applied a primary coating of DOPA on PCL surfaces, followed by grafting AL on the DOPA coatings using genipin (GP) crosslinking as a secondary coating. This modified PCL scaffold was referred to as D-PCL@AL. The authors demonstrated that D-PCL@AL partially promoted bone regeneration in bone defect models by activating the Piezo1-YAP-TG2 axis 172. Bioelectricity is indispensable in cell division, intracellular communication, neuronal activities, and ion transport in living systems 173. Piezoelectric biomaterials have raised much attention in tissue regeneration because they can generate electrical activity under deformation stimulation without requiring an external power source 173. Kaliannagounder and colleagues developed a piezoelectric whitlockite (Ca18Mg2(HPO4)2(PO4)12) nanoparticles (WH NPs) to mimic bioelectric activity and enhance tissue regeneration 174. They discovered that piezoelectric WH nanoparticles could promote osteogenic differentiation of MC3T3E1 cells w/o LIPUS stimulation *in vitro*, likely due to the activation of Piezo1 and TRPV4 channels 174. In addition, piezoelectric micro-vibration stimulation (PMVS) inhibits bone resorption and promotes osteogenic differentiation, improving trabecular morphology in ovariectomized (OVX) mice through activating Piezo1, miR-29a and Wnt signaling 175.

Pharmacological activation of Piezo1 by Yoda1 has been reported to promote osteogenesis and angiogenesis 46. Yoda1 mimics the beneficial effect of mechanical loading on bone cells 75. Interestingly, Yoda1 can significantly increase bone mass without affecting the body weight of mice *in vivo* 75. Yang *et al.* fabricated a Yoda1 loaden membrane with inner and outer layers. The inner layer is responsible for bone regeneration by controlled release of Yoda1, promoting cell proliferation and osteogenic differentiation. Simultaneously, the outer layer acts as a barrier to prevent infiltration of fibrous connective tissue into the bone defect area. The researchers found that the Yoda1 bilayer membrane promotes bone healing via the Piezo1/RhoA/ROCK1/YAP1 signaling pathway 176. Kong *et al.* developed a TiO2 Nanotube and found that TiO2 nanotubes promote osteogenesis both *in vitro* and *in vivo* via the Piezo1-Yap1 signaling pathway 177. Wang* et al.* fabricated a wearable pulsed triboelectric nanogenerator (WP-TENG) that generates electric flow during human body movement 178. The WP-TENG enhances osteogenic differentiation and proangiogenic functions of aged BMSCs by Piezo1 activation* in vitro*, leading to increased Ca^2+^ influx and regulation of HIF-1α transcriptional activity. Eventually, this results in the upregulation of osteogenic markers (Col1a, Runx2, and OCN) and angiogenic markers (EDN1 and VEGFA). *In vivo*, the WP-TENG activates aged BMSCs to enhance bone defect repair and regeneration in a Piezo1-dependent manner. Chen *et al.* have identified that intermittent exposure to extremely low-frequency pulsed electromagnetic fields (ELF-PEMF) showed better effects on the maturation of osteoprogenitor cells than continuous ELF-PEMF, and this effect was mediated by increased Piezo1 expression 67. Besides, a 3D stiff matrix activates Piezo1/AMPK/autophagy in MC3T3-E1 cells, contributing to osteogenic processes 179.

### Osteoarthritis and Joint Health

OA refers to the most common joint disease worldwide, affecting approximately 9.6% of men and 18% of women aged over 60 180. It is the major source of pain, disability, and decreased quality of life in aging individuals 181. Chronic overload and resultant inflammation are key factors in OA progression 181. Currently, the treatment strategies of OA primarily focus on pain management and joint replacement for end-stage OA 182. The mechanosensitive ion channel, Piezo1, is expressed in chondrocytes and is involved in the pathogenesis of OA 53. Study has shown that higher expression of Piezo1 was found in osteoarthritic cartilage compared with healthy human cartilage 183. Cytoskeleton proteins are key players in mechanical signal transduction during OA 184. Piezo1 regulates the polymerization and depolymerization of the cytoskeleton 185, and the cytoskeleton reduces Piezo1 activation and vice versa 183, 186, 187, suggesting that Piezo1 is closely associated with the cytoskeleton 184. Inhibition of Piezo1 can reduce the abnormal proliferation of chondrocytes caused by excessive mechanical loading. This is closely related to cytoskeleton protection 183, 187.

Chondrocyte ferroptosis, a form of cell death during degeneration and aging, has been identified as a crucial factor in the pathogenesis of OA 188. Recent research has highlighted the role of Piezo1 in this process. Studies have shown that mechanical overloading results in human chondrocyte ferroptosis mediated by the Piezo1 channel 189. Activation of Piezo1 by Yoda1 is associated with increased dead cells in chondrocytes under excessive loading stimulation, along with a decrease in glutathione peroxidase 4 (GPX-4), a marker of cell ferroptosis 189. Inhibiting ferroptosis by injecting ferrostatin-1 (Fsp1) could attenuate OA progression 190. Furthermore, Zhao *et al*. found that GPX-4 knockout impairs the basic response to mechanical overloading, suggesting the importance of GPX-4 in protecting against ferroptosis in chondrocytes 189.

Inflammatory factors, for example, IL-1α, secreted by chondrocytes during the development of OA, have been shown to enhance the sensitivity of Piezo1-related pathways, making chondrocytes more susceptible to mechanical overloading 183. This increased vulnerability can be attributed to a pathogenic feed-forward signaling mechanism involving p38 MAP-kinase and transcription factors hepatocyte nuclear factor 4 (HNF4) and activating transcription factor 2 (ATF2) /CREBP1 183. In addition, IL-1β can upregulate the expression of Piezo1 in human chondrocytes and then inhibit chondrocyte autophagy and enhance chondrocyte apoptosis through the PI3K/AKT/mTOR pathway 191. Static magnetic field (SMF) promotes MSCs migration and chondrogenic differentiation to enhance cartilage repair and alleviate OA symptoms 192. Piezo1-induced CXCR4 is further identified as key mechanism behind SMF-enhanced MSC recruitment and subsequent repair 192.

Heterochromatin instability, a defining characteristic and influential factor in senescence, plays a crucial role in the regulation of the senescence-associated secretory phenotype, which triggers inflammation and leads to cartilage damage 111. AURKB, a vital component of the chromosomal passenger complex, is associated with the destabilization of heterochromatin 193. Ren *et al.* have demonstrated that mechanical overloading increases in AURKB levels via the Piezo1 channel. Interestingly, the utilization of Barasertib, an inhibitor of AURKB, has shown the potential to enhance heterochromatin stability and reduce chondrocyte senescence, thereby alleviating the progression of OA 111. Additionally, mechanical overloading also increases miR-155-5p expression and reduces GDF6 through Piezo1 activation. However, the administration of exogenous GDF6 has been found to attenuate OA progression by activating SMDA2/3 phosphorylation 194.

The G protein-coupled estrogen receptor (GPER) inhibits Piezo1 expression by reducing actin polymerization and inhibiting the RhoA/LIMK/cofilin pathway 195. Additionally, Yoda1 aggravates OA progression while Artemisinin (ART) protects cartilage from damage by inhibiting the Piezo1-PI3K-AKT signaling pathway 196. Of note, the role of Piezo1 in OA alleviation remains controversial. Young *et al*. found that *Piezo1* and *Piezo2* conditional knockout (*Piezo1^Gdf5Cre^; Piezo2^Gdf5Cre^*) cannot protect cartilage from injury 197. However, a recent study by Laura *et al.* found that inactivation of *Piezo1* in chondrocytes (*Piezo1^Col2a1Cre^*) alleviates cartilage degeneration and osteophyte formation following OA 198. The different results may be attributed to the type of mice used, gene ablation efficiency, and surgery methods. Although, *Ptgs2*, and *Ccn2* are identified as potential downstream genes of Piezo1 in chondrocytes 198, the molecular mechanism of the role of Piezo channels in cartilage still needs to be further investigated.

### Muscle Atrophy and Regeneration

Muscle mass depends on the balance of protein synthesis and degradation. A reduced muscle mass impedes the body response to stress stimulation and chronic disease 117. In clinical situations, Duchenne muscular dystrophy (DMD) represents a life-threatening genetic neuromuscular disorder that leads to progressive muscle weakness in skeletal and cardiac muscle 199. Improper MuSC activation exits during the progression of DMD 200. In dystrophic mice, more “sensory” cells and less “responsive” cells are located in dystrophic muscles along with Piezo1 reduction 130, reactivation of Piezo1 by Yoda1 improves the function of dystrophic MuSCs by shortening the protrusion length and enhancing MuSCs function upon repetitive injuries 130. Skeletal muscle adapts to reduced activity by undergoing atrophy. Immobilization is one of the most common reasons for muscle atrophy 201. During immobilization, the diminished influx of Ca^2+^ acts as one of the contributing factors to the initiation of skeletal muscle atrophy 202. Mice hind limb immobilization in a cast for three days results in a 10% to 15% decrease in muscle mass 202. Previous evidence found that Piezo1 activation enhances the fusion index of myotubes 54. Recent evidence showed that Piezo1 inhibition by GsMTx-4 increases muscle atrophy-related genes (*Klf15* and *Il6*), and Piezo1 activation by Yoda1 reduces the expression of *Klf15* and *Il6* 202. The atrophy-related genes were upregulated in skeletal muscle conditional knockdown *Piezo1* mice 202. Clinical samples harvested from the patients who suffered cast immobilization after bone fracture had less *Piezo1* gene expression in muscles 202. Thus, we suggest that Piezo1 is involved in regulating skeletal muscle atrophy.

### Intervertebral Disc Degeneration

IDD, a type of chronic skeletal disease, is one of the common causes of low back pain. Excessive mechanical stimulation induces apoptosis and senescence of NP and AF cells, which play critical roles in the development of IDD 203. NP-like differentiation of stem cells is essential for IDD regeneration. Huang *et al.* fabricated injectable upper critical temperature (UCST) microgels to measure the effects of static stretch by swelling microgels on stem cell fate determination 204. They found that UCST microgels combined with adipose-derived mesenchymal stem cells (ADSCs) promoted NP-like differentiation of stem cells as enhanced by Piezo1 and TRPV4 activation *in vitro*. *In vivo*, ADSCs-loaded UCST microgels injection increased ECM production and water content, suggesting that mechanical stimulation produced by injectable microgel may be an effective approach for IDD repair 204.

## Conclusion and Perspectives

Piezo1, as a key mechanosensitive ion channel, holds great promise in unraveling the intricate mechanisms underlying musculoskeletal physiology and pathology. In summary, the high expression of Piezo1 in different types of cells in the musculoskeletal system highlights its importance on bone formation, OA progression, myotube formation, tendon stiffness, and AF and NP cell apoptosis and senescence. However, the understanding of thePiezo1's role in the musculoskeletal system is still in its infancy. Most of the studies just focus on phenotypic changes, such as the direct effects of Piezo1 activity on stem cell function. Besides, Piezo1 may enhance other events, like CGRP release and angiogenesis, to guide stem cell behaviors indirectly. In addition, research needs to be conducted in other tissues within the musculoskeletal system, such as ligament and tendon-bone-junction. More importantly, Piezo1 plays an essential role in the regulation of physical activity, which is essential for aging people with musculoskeletal disorders. Exploring ways to enhance physical performance via regulating Piezo1 expression is an important avenue for investigation. Most recently, bioinformatics analysis showed that some key genes, such as *Lcn2*, *Dkk3*, and *Tnnt1* are negatively associated with Piezo1. However, the exact relationship between Piezo1 and these genes-related pathways is still unclear. Manipulation of Piezo1 activity by drugs and mechanical stimulation will likely be developed as a new useful strategy to treat musculoskeletal disorders. Although GsMTx4 benefits OA and IDD, it is not specific to Piezo1. More investigations are needed to resolve the molecular structure of Piezo1 to fabricate additional modulators.

## Figures and Tables

**Table 1:** Different types of physical stimulations on cell fate determination via Piezo1 in the musculoskeletal system

Cell type	Mechanical stimulation	Parameters	Frequency	Device	Outcomes	Mechanism
Aged human BMSCs 178	Wearable pulsed triboelectric nanogenerator (WP-TENG)	30 µA	30 min/day, 7 days in total	Fabricated by a nylon sheet, foam paper, Cu foil, Al foil, PTFE film, petri dish, and needle	Osteogenesis↑; WP-TENG treated BMSCs for 1 week enhance tube formation	Rejuvenation of Aged BMSCs by the activation of mechanosensitive ion channel Piezo1, stimulating Ca^2+^ influx, and regulating HIF-1α. Thus, osteogenic and angiogenic markers elevate.
Mouse BMSCs 77	Cyclic tensile strain	0.5 Hz	0%, 3%, 8%, 13%, and 18%, 8 h/day for 3 days	FX-4000TM Tension System (Flexcell International Corporation)	Osteogenesis↑	Mechanical stretch promotes osteogenic differentiation via mechanosensitive ion channels (TRPV4, Piezo1, and Piezo2).
Mouse BMSCs 192	SMF	0, 50, 100, and 200 mT	2 weeks	Generated by a set of permanent magnets (35 mm in diameter and 10 mm in thickness)	Migration↑; Chondrogenic marker (Col2, and Sox9↑); MMP13↓	SMF activates SDF-1/CXCR4 signaling pathway through Piezo1
Human UE7T-13 cells 20	HP	0.01 MPa	10 days	Custom-made pressure chamber	Piezo1, and BMP2↑; osteogenesis↑; adipogenesis↓	Piezo1 acts as a receptor for HP and functions at the branch points of cell fate decisions of MSCs by regulating BMP2 expression.
Mouse MC3T3-E1 cells 47	FSS	100-120 rpm (about 2 Pa at the edge)	1 h	Horizontal shaking apparatus	Piezo1 protein↑; Runx-2 gene↑	Piezo1 induced by FSS activates AKT/GSK-3β/β-catenin pathway following the phosphorylation of Akt and phosphorylation of GSK-3β. Then the β-catenin translocates to the nucleus to modulate Runx-2 gene expression.
MC3T3-E1 78	LFF	10 dynes/cm^2^	1 h	FlexCell; STR-4000	Ptgs2 and Serpine1 expression↑	LFF induces the expression of mechanoresponsive genes via Piezo1 activation.
MC3T3-E1 17	LIPUS	1-Hz pulse repetition frequency, 20% duty cycle, 200-mV amplitude, and 2.25-MHz burst sin wave and amplified by a radio-frequency power amplifier	3 and 6 min	Generator: AFG3021, Tektronix Inc, Beaverton, OR; Amplifier: E&I 2100 L, Electronics & Innovation, Ltd., Rochester, NY; Transducer: Shinjuku, Tokyo, Japan	MC3T3-E1 migration and proliferation	LIPUS activates ERK1/2 phosphorylation and perinuclear F-actin polymerization via Piezo1 activation.
Mouse MC3T3-E1 175	PMVS	120 Hz	1 h	Microchip pulse generator (Microchip Technology Inc. Chandler, AZ, USA), a micro-amplifier (Texas Instruments, Dallas, TX, USA), a pulse wave modulator (Texas Instruments, Dallas, TX, USA), and 6 ceramic piezoelectric vibration transducers (1-cm in diameter; Steiner & Martins, Inc., Davenport, FL, USA)	Piezo1 gene and miR-29a gene↑; Osteogenic markers (Runx2, Ocn expression↑)	PMVS activates Piezo1 signaling, thus, increasing Wnt and miR-29a signaling to promote osteoblastic activity.
Mouse MC3T3-E1 174	WH NPs and LIPUS	LIPUS: 1 MHz, 0.3 W/cm^2^, and 20% (pulsed ratio 1:4)	WH: 100 μg/ml for 24 h; LIPUS :20 min daily at 37 ^o^C	Custom-made WH NPs	Osteogenic markers (Runx2, Ocn, Opn, Piezo1 and TRPV4 expression↑)	WH NPs promotes osteogenic differentiation via increasing Piezo1 and TRPV4 mRNA level.
Mouse MC3T3-E1 81	FSS	12 dynes/cm^2^	2 h	Generated by the patch-clamp recording at an angle of 80^o^ using a fire-polished glass pipette (tip diameter 3-4 mm)	Piezo1 and osteogenic maker (alp, bglap, and Col1α1)↑	Piezo1 senses FSS and consequently regulates its expression, osteoblast function and bone formation.
Murine MLO-Y4 (osteocytes) 50	FSS	2, 4, 8, and 16 dynes/cm^2^	10 min	Created by parallel plate flow chambers separated by a gasket of defined thickness with gravity-driven fluid flow using a peristaltic pump	Activate Piezo1, enhance the colocalization of Piezo1 and Cx43 HCs, and induce the opening of Cx43 HCs	Piezo1 activation by FSS further activates Cx43 HCs and Panx1 channels through activating the PI3K-Akt signaling pathway.
Mouse MLO-Y4 osteocytes 87	FSS	9 dynes/cm^2^	30 min	Flow loop apparatus 205	Piezo1 protein, OPG expression↑; NOTCH3, and RANKL↓	Piezo1 mediated FSS promotes the expression of OPG and inhibits the expression of RANKL via NOTCH3.
Murine IDG-SW3 cells (osteocytes) 48	Mechanical stretch	5 Hz, Stretch ratio: 5%	continuous cyclic stretching for 10, 30, 60, 120, 180 min	ShellPa Pro, Menicon Life Science	Rapid Ser473 phosphorylation of Akt; *Sost* gene↓	Mechanical stimulation of osteocytes suppresses *Sost* expression via the Piezo1-Akt pathway.
Murine RAW264.7 100	Mechanical stretch	cyclic sinusoidal continuous tensile strain (10%, 0.5 Hz)	2 h	Flexcell® FX-5000™ Tension System (Flexcell International Corporation)	M2-type macrophage transformation↑; Macrophage-derived medium enhance proliferation, migration, and osteogenic differentiation of BMSCs	Mechanical tension causes calcium influx, p53 deacetylation, macrophage polarization towards M2 and TGF-β1 release through Piezo1.
Human SCP-1 cells 67	Extremely low frequency pulsed electromagnetic fields (ELF-PEMF)	16 Hz	10 min exposure every 8 h for 3 days	Somagen®, CE 0482, compliant with EN ISO 13485: 2016 + EN ISO 14971: 2012	Osteogenic differentiation↑, Piezo1 expression ↑	Intermittent exposure ELF-PEMF promotes maturation of osteoprogenitor cells mediated by increased Piezo1 expression.
Human chondrocytes 195	Cyclic stress	6 cycles per minute with 20% surface elongation	24 h	Multi-channel stress loading system FX-4000T (Flexercell International, McKeesport, USA)	Apoptosis↑; actin polymerization↑; Piezo1 activation	NA
Human chondrocytes 187	Cyclic stress	10 cycles per minute with 20% surface elongation	0 h, 2 h, 12 h, 24 h, 48 h	Multichannel cell stretch stress loading system FX-4000T (Flexcell, USA)	Piezo1 activation; Kif8a gene and protein↑	Mechanical stretch activates Piezo1, resulting in the overexpression of Kif8a, which leads to microtubule depolymerization, destroys the integrity of cytoskeleton, and inhibits the mitosis of cells.
Rat chondrocytes 110	Excessive mechanical strain	20% elongation, 0.1 Hz frequency	24 h	Flexcell Tension System (FX4000T; FlexCell International Corporation, Burlington, NC, USA)	Apoptosis↑; anabolic and catabolic imbalance	Excessive mechanical strain induces apoptosis and the anabolic and catabolic imbalance via CaN/NFAT1 Signaling Axis mediated by Piezo1.
Mouse chondrocytes 189	Excessive mechanical strain	1 MPa, at a frequency of 1 Hz	1 h	Pneumatic component (FESTO, Germany)	GPX4 gene and protein↓; ROS production↑	Mechanical overloading induces ferroptosis in chondrocytes through the Piezo1 channel
Mouse myoblasts 54	Mechanical strain	After an initial 1 min rest period (0% stretch), stretch is applied at 3% (0.3 mm), 6% (0.6 mm), and 9% (0.9 mm) for 1 min, followed by rest for 1 min in between		Modified elastic silicone chambers (Strexcell, Ooyodonaka, Reference; STB-CH-0.02)	Piezo1 activation; myotube formation↑	Piezo1 expression and activity is crucial for Ca^2+^ regulation in muscle function.
Human NP cells 57	Cyclic stress	6 cycles per minute with 20% surface elongation	6 h, 12 h, and 24 h	Multi-channel stretch loading system FX-4000T (Flexercell International, McKeesport, USA)	Piezo1 and NLRP3 inflammasome activation;	Mechanical stretch activates Piezo1-dependent NLRP3 inflammasome via NF-κB pathway.
Human NP cells 60	Mechanical stress	1.0 Hz with 15% or 1.5% compression intensity	12 h, 24 h, and 48 h	Flexcell FX5000 Compression system (Flexcell International, McKeesport, USA)	Piezo1 gene and protein↑; inflammatory cytokine (TNF-α, IL-6, and IL-1β) ↑; mitochondrial damage; senescence marker (p53 and p16)↑	Excessive mechanical stress promotes the apoptosis, senescence, and proinflammatory cytokines of NP cells via Piezo1 activation.
AF cells 58	Cyclic stress	0.5 Hz, 5-20% stretch deformation	36 h	Flex-Cell 5000 tension system (Flexcell International)	AF cells apoptosis↑; Piezo1 activation	Excessive mechanical loading promotes AF cell senescence via Piezo1/Ca^2+^/Calpain2/Caspase3 pathway
Human NP cells 143	Mechanical stress	Cell shape variable was 15%, deformation period 20 s	24 h	Flexcell mechanical distraction stress loading instrument (Flexcell® International Corporation, Burlington, VT)	NP cells apoptosis↑; Piezo1 activation	Excessive mechanical stress promotes the apoptosis of NP cells via Piezo1 activation.

NP cells, nucleus pulposus cells; AF cells, annulus fibrosus cells; FSS, fluid shear stress; SMF, static magnetic field; LIPUS, low-intensity ultrasound stimulation; HP, hydrostatic pressure; LFF, laminar fluid flow; PMVS, piezoelectric microvibration stimulation; BMSCs, bone marrow stromal cells; HIF-1α, Hypoxia-inducible factor 1-alpha; Col2, Collagen type 2; Sox9, SRY-Box Transcription Factor 9; Runx2, Runt-related transcription factor 2; Ocn, osteocalcin; OPN, osteopontin; MMP13, matrix metalloproteinase 13; SDF-1, stromal-cell derived factor-1; CXCR4, C-X-C Motif Chemokine Receptor 4; BMP2, Bone Morphogenetic Protein 2; GSK-3β, Glycogen synthase kinase-3 beta; Ptgs2, Prostaglandin-Endoperoxide Synthase 2; and Serpine1, Serpin Family E Member 1; ERK1/2, The extracellular signal-regulated kinase 1/2; Cx43 HCs, connexin 43 hemichannels; Panx1, Pannexin 1; NOTCH3, Neurogenic locus notch homolog protein 3; RANKL, Receptor activator of NF-kB ligand; PI3K-Akt, TGF-β1, Transforming growth factor beta 1; Kif8a, Kinase-like protein 18A; NFAT1, nuclear factor of activated T cells 1; OPG, osteoprotegerin; NLRP3, Nod-like receptor protein 3; NA, not applicable.

## Abbreviations

AS: ankylosing spondylitis
AL: alendronate
ART: artemisinin
ADSCs: adipose-derived mesenchymal stem cells
AF: annulus fibrosus
BMD: bone mineral density
BMSCs: bone marrow mesenchymal stromal cells
Cx43 HCs: connexin43 hemichannels
COMP: cartilage oligomeric matrix protein
CEP: cartilaginous endplate
CaN: calcineurin
DOPA: dihydroxyphenylalanine
DMP: dentin matrix protein 1
DMD: Duchenne muscular dystrophy
ER: endoplasmic reticulum
ECs: endothelial cells
FSS: fluid shear stress
Fsp1: ferrostatin-1
GsMTx4: grammostola spatulata mechanotoxin 4
Gd^3+^: gadolinium
GPX-4: glutathione peroxidase 4
GPER: G protein-coupled estrogen receptor
GP: genipin
HNF4: hepatocyte nuclear factor 4
HP: hydrostatic pressure
IFP: infrapatellar fat pad
IDD: intervertebral disc degeneration
LIPUS: low-intensity ultrasound stimulation
LEPR: leptin receptor
MCs: myeloid-lineage cells
MuSCs: muscle stem cells
MSCs: mesenchymal stem cells
NFAT1: nuclear factor of activated T cells 1
NLRP3: nod-like receptor protein 3
NO: nitric oxide
NP: nucleus pulposus
OA: osteoarthritis
OP: osteoporosis
Oln: osteolectin
OVX: ovariectomized
OPG: osteoprotegerin
PC2: polycystin-2
PSCs: periosteal stem cells
PEMF: pulsed electromagnetic fields
PCL: polycaprolactone
PS: phosphatidylserine
Pifithrin-α: PFT-α
PMVS: piezoelectric micro-vibration stimulation
RR: ruthenium red
SERCA: sarco/endoplasmic reticulum Ca^2+^-ATPase
SM: synovial membrane
SNPs: single nucleotide polymorphisms
SIBLING: small integrin-binding ligand N-linked glycoprotein
SMF: static magnetic field
TFRC: transferrin receptor
Ucn: urocortin
WP-TENG: wearable pulsed triboelectric nanogenerator
5-HT: serotonin
